# Molecular identification, genotyping of virulence-associated genes, and pathogenicity of cellulitis-derived *Escherichia coli*

**DOI:** 10.14202/vetworld.2020.2703-2712

**Published:** 2020-12-19

**Authors:** Mohamed M. Amer, Hoda M. Mekky, Hanaa S. Fedawy, A. EL-Shemy, M. A. Bosila, Kh. M. Elbayoumi

**Affiliations:** 1 Department of Poultry Diseases, Faculty of Veterinary Medicine, Cairo University, P.O. 12211, Giza, Egypt; 2 Poultry Diseases Department, Veterinary Research Division, National Research Centre, P.O. 12622, Giza, Egypt; 3 Department of Parasitology and Animal Diseases, Veterinary Research Division, National Research Centre, P.O. 12622, Giza, Egypt

**Keywords:** avian pathogenic *Escherichia coli*, cellulitis, colibacillosis, polymerase chain reaction, virulence-associated genes

## Abstract

**Background and Aim:**

Avian colibacillosis, which is caused by avian pathogenic *Escherichia coli* (APEC), is a major bacterial disease that affects birds of all ages worldwide, causing significant economic losses. APEC manifests in several clinical forms, including cellulitis, and its high pathogenicity is attributed to harboring numerous virulence-associated genes (VGs). This study evaluated the pathogenicity of the cellulitis-derived *E. coli* (O78) strain through molecular identification of genes coding for seven virulence factors and by conducting an *in vivo* assessment of capability for cellulitis induction in broiler chickens.

**Materials and Methods:**

This study was performed using a previously isolated and identified cellulitis-derived *E. coli* (O78), which was screened for seven VGs using molecular detection and identification through polymerase chain reaction followed by nucleotide sequencing and phylogenetic analysis. Experimental infection by subcutaneous (SC) inoculation in broilers and its pathogenicity was confirmed *in vivo* by cellulitis induction. The impact of cellulitis on broiler performance was assessed.

**Results:**

Molecular genotyping proved that the isolate harbored five virulence genes (*iro*N, *iut*A, *tsh*, *iss*, and *pap*C) and was negative for *stx*1 and *hly* genes. The amplified products for *iro*N, *iss*, and *iut*A were subjected to sequencing and phylogenetic analysis, and the results indicate the highest similarity and matching with *E. coli* submitted to the National Center for Biotechnology Information GenBank. SC inoculation of bacteria in broiler chickens resulted in cellulitis, as indicated by thick red edematous skin with yellowish-white material in the SC tissue at the inoculation site, and the abdominal muscle showed redness and increased vacuolization. Histopathological examination revealed moderate-to-severe caseous inflammatory reaction with a marked accumulation of heterophils and mononuclear cells in the SC fatty tissue. The average feed intake, body weight gain (BWG), and feed conversion ratio (FCR) were lower in infected chickens in comparison with those of the control non-infected chickens.

**Conclusion:**

This study proves that molecular techniques are accurate for pathogenicity determination in virulent bacteria, with the advantages of being rapid, time-saving, and economical. Cellulitis is associated with economic losses that are represented by a lower BWG and FCR.

## Introduction

Avian colibacillosis is an infectious disease affecting birds of all ages. It is a major bacterial disease that adversely impacts the poultry industry, resulting in significant economic losses and welfare concerns worldwide [1,2]. Economic losses are attributed to the high costs of treatment and vaccination, lower growth rate and egg production, high mortality, and carcass condemnation at slaughterhouses [2]. The disease is caused by avian pathogenic *Escherichia coli* (APEC), which is a sub-pathotype of the extraintestinal pathogenic *E. coli*, with zoonotic potentiality [2,3]. Colibacillosis is a complex syndrome that can manifest as a localized or systemic infection, such as yolk sac infection, respiratory tract infection, swollen head syndrome, septicemia, polyserositis, coligranuloma, enteritis, salpingitis, pericarditis, perihepatitis, and cellulitis [2,4].

One of the most important forms of avian colibacillosis is avian cellulitis (AC), which results from subcutaneous (SC) invasion of bacteria. AC has emerged as an economically significant disease syndrome in broiler chickens due to increased condemnation, downgrading at processing, and increased costs associated with the trimming and reprocessing of affected carcasses [5]. AC is characterized by a diffuse spreading edematous with suppurative inflammation of the deep SC tissues, sometimes extending into the muscle and frequently associated with abscess formation, commonly referred to as “plaques” [6]. Clinically, the affected birds appear normal, and lesions are not detected until processing [7]. AC is caused by numerous bacterial species, but APEC remains the most frequently isolated bacteria [6,8-10], while *E. coli* isolated from cellulitis lesions were more likely to induce cellulitis lesions in experimentally infected birds than in non-cellulitis-derived strains [11].

The high pathogenicity of APEC is attributed to harboring numerous virulence-associated genes (VGs) that enable bacteria to invade, colonize, and evade the immune system, thus causing an extraintestinal form of avian colibacillosis. Many virulence-associated factors were expressed in *E. coli* strains that had been isolated from cellulitis and other colibacillosis lesions [12,13]. A study suggested that eight VGs–P-fimbriae (*pap*C); aerobactin (*iuc*D); iron repressible protein (*irp*2); temperature-sensitive hemagglutinin (*tsh*); vacuolating autotransporter toxin (*vat*); enteroaggregative toxin (*ast*A); increased serum survival protein (*iss*); and colicin V plasmid operon genes (*cva/cvi*) – contributed to the pathogenicity of APEC. The authors proposed that the presence of four of these eight VGs could identify APEC [14]. De Carli *et al*. [15] reported the pathogenicity of the APEC strain in the presence of five VGs. Recently, pathogenicity has been evaluated by the existence of at least three VGs, according to the genetic criteria, which enable them to survive an extraintestinal life [16,17]. Another study, carried out in Brazil [18], recorded a positive association between the number of VGs and the pathogenicity score of avian colibacillosis. Authors found that 51% of the isolated APEC harbored an assortment of VGs, including adhesions, iron uptake systems, increased serum survival, and toxins. In addition to the pathogenicity determination, some studies reported that APEC-related VGs could be used as molecular markers for the detection and identification of APEC strains [3,14].

This study aimed to molecularly evaluate the pathogenicity of a cellulitis-derived *E. coli* (O78) strain through the identification of genes coding for seven virulence-associated factors, as well as *in vivo* assessment of the pathogen’s capability to induce lesions in broiler chickens.

## Materials and Methods

### Ethical approval

This study was approved by the Ethical Committee for Medical Research at the National Research Centre, Egypt, and in accordance with local laws and regulations.

### Study period and location

This study was conducted from September to November 2019 at the Department of Poultry Diseases, Faculty of Veterinary Medicine, Cairo University and Poultry Diseases Department, Veterinary Research Division, National Research Centre, Giza, Egypt.

### Bacterial strain

This study was carried out using the purified culture of cellulitis-derived *E. coli* (O78). The predominant strain was originally isolated, purified, and identified from cellulitis lesions in broiler chickens [10]. Bacteria were cultured in a liquid broth, as described previously [19], and screened for VGs through the molecular detection and identification of seven genes. These seven genes are APEC-associated virulence genes that are commonly detected in the majority of studies that have focused on APEC [20-24].

### Polymerase chain reaction (PCR)

#### Bacterial DNA extraction

DNA extraction from samples was performed using the QIAamp DNA Mini Kit (Qiagen, Germany, GmbH), with modifications to the manufacturer’s recommendations. Briefly, 200 μL of the culture broth was incubated with 10 μL proteinase K and 200 μL lysis buffer at 56°C for 10 min. After incubation, 200 μL absolute ethanol (100%) was added to the lysate. Then, the sample was centrifuged and washed following the manufacturer’s recommendations, and nucleic acid was eluted with 100 μL of the elution buffer that was provided in the kit.

### Oligonucleotide primers

The used primers used for this study were supplied from Metabion (Germany) and are listed in Table-1.

**Table-1:** Oligonucleotide primers sequences of target *Escherichia coli* genes with amplicon sizes and cycling conditions.

Target gene	Primers sequences (5’- 3’)	Amplified segment (bp)	Prim. Den.	Amplification (35 cycles)			Final extension	References

				Sec. den.	Ann.	Ext.		
*iss*	F ATGTTATTTTCTGCCGCTCTG R CTATTGTGAGCAATATACCC	266	94°C 5 min	94°C 30 s	54°C 30 s	72°C 30 s	72°C 7 min	Yaguchi *et al*.[25]
*iut*A	F GGCTGGACATGGGAACTGG R CGTCGGGAACGGGTAGAATCG	300	94°C 5 min	94°C 30 s	63°C 30 s	72°C 30 s	72°C 7 min	
*hly*	F AACAAGGATAAGCACTGTTCTGGCT R ACCATATAAGCGGTCATTCCCGTCA	1177	94°C 5 min	9°C 30 s	60°C 40 s	72°C 1 min.	72°C 12 min.	Piva *et al*.[26]
*iro*N	F ATCCTCTGGTCGCTAACTG R CTGCACTGGAAGAACTGTTCT	847	95°C 5 min	94°C 30 s	50°C 40 s	72°C 50 s	72°C 10 min.	Ewers *et al*.[27]
*tsh*	F GGTGGTGCACTGGAGTGG R AGTCCAGCGTGATAGTGG	620	95°C 5 min	94°C 30 s	54°C 40 s	72°C 45 s	72°C 10 min.	Delicato *et al*.[28]
*pap*C	F TGATATCACGCAGTCAGTAGC R CCGGCCATATTCACATAA	501	95°C 5 min	94°C 30 s	58°C 40 s	72°C 45 s	72°C 10 min.	Wen-jie *et al*.[29]
*stx*1	F ACACTGGATGATCTCAGTGG R CTGAATCCCCCTCCATTATG	614	95°C 5 min	94°C 30 s	58°C 40 s	72°C 45 s	72°C 10 min.	Dipineto *et al*.[30]

### PCR amplification

Primers were utilized in a 25 μL reaction containing 12.5 μL Emerald Amp Max PCR Master Mix (Takara, Japan), 1 μL of each primer (20 pmol concentration), 4.5 μL water, and 6 μL of DNA template. The reaction was performed in an Applied Biosystems 2720 Thermal Cycler. The amplification conditions and amplified product are listed in Table-1 [25-30].

### PCR product analysis

The products from the PCR were separated by electrophoresis on 1.5% agarose gel (Applichem, Germany, GmbH) in 1× TBE buffer at room temperature using gradients of 5 V/cm. For the gel analysis, 15 μL of the amplified products was loaded in each gel slot, and GelPilot 100 bp Plus DNA Ladder (Qiagen, Germany, GmbH) was used to determine the fragment sizes. The gel was photographed by a gel documentation system (Alpha Innotech, Biometra), and the data were analyzed with computer software.

### Gene sequence

PCR products were purified using QIAquick PCR Product Extraction Kit (Qiagen, Valencia, CA, USA). BigDye Terminator V3.1 Cycle Sequencing Kit (PerkinElmer, Foster city, CA, USA) was used for the sequence reaction, and then, the product was purified using Centri-Sep™ spin columns. DNA sequences were obtained from Applied Biosystems 3130 genetic analyzer (Hitachi, Japan). A BLAST^®^ analysis (Basic Local Alignment Search Tool) was initially performed to establish sequence identity to GenBank accessions [31]. A phylogenetic tree was created using the CLUSTAL W multiple sequence alignment program, MegAlign module of Lasergene DNASTAR version 12.1 [32].

### Phylogenetic analyses

Phylogenetic analyses were performed using the maximum likelihood, neighbor-joining, and maximum parsimony in MEGA6 [33].

### Experimental infection

#### Chickens

Forty-one-day-old Ross 308 broiler chickens were obtained from a commercial hatchery immediately after hatching. This hatchery is recognized to have no history of *E. coli* for a long time period. Immediately after arrival of the chickens, a random representative number of chickens were examined bacteriologically. All birds were negative for *E. coli* on cultural examination. The chickens were reared on deep litter.

#### Ration

The chickens were fed on commercial rations according to the nutritional requirements of chickens [34] and given pelleted starter (crude protein not <23%) and growing (crude protein not <21%) rations. Drinking water and rations, without feed additives, were given to the chickens *ad libitum*.

#### Vaccination

All chickens received the ND+IBV vaccine on the 5^th^ day of age, IBD intermediate 228E on the 10^th^ day, and La Sota on the 16^th^ day. All vaccines were administered through ocular instillation.

#### Inoculum preparation

*E. coli* O78 isolate was cultured and propagated as described previously [19] and was used at a concentration of 10^8^ colony-forming units/mL.

#### Experimental infection

At 14 days old, chickens were randomly divided into two groups (20 birds/ group). Group 1 was kept as non-infected (the negative control group) and Group 2 (the challenge group) was injected (SC) in the thigh fold with 1 ml *E. coli*. Infected birds were observed daily, and clinical abnormalities and mortalities were recorded by examination of the inoculation site. In addition, the daily body weight gain (BWG), feed intake, and feed conversion ratio (FCR) were recorded on 7, 14, and 19 days of age to ensure that the applied management procedures are optimum and to exclude extra factors (like stress or other infectious agents) that may deteriorate these parameters so that abnormalities or modifications in these parameters will be solely attributed to our induced treatment (cellulitis). Tissue samples (skin section) were collected and fixed in 10% neutral buffered formalin for histopathological examination.

### Broiler performance parameters

The FC and conversion ratio were determined using the following formula: FC g/bird=Feed intake in a replication/No. of live birds in a replication. FCR=Feed intake (g)/Live weight (g). Parameters were recorded for each chicken in both groups on 7, 14, and 19 days of age, according to the NRR [34].

### Tissue specimens for histopathological examination

Skin specimens were collected on 3- and 5-day post-infection (DPI) and fixed in 10% neutral buffered formalin to prepare the paraffin tissue sections at 4–6 μm thickness. These sections were stained with hematoxylin and eosin [35].

### Statistical analysis

Performance data were compared using the mixed model analysis of variance and a compound symmetry covariance matrix (PROC MIXED, SAS 9.2, SAS Inc., Cary, NC). p<0.05 or p<0.001 was considered to be significant. The obtained results revealed that the differences among the compared data were non-significant.

## Results and Discussion

The pathogenicity of APECs is attributed to the interactions among several virulence-associated factors, such as adhesion capacity, colicin production, presence of aerobactin, serum resistance, temperature-sensitive hemagglutinin, and presence of certain capsular antigens [28]. Different APEC strains may have unique combinations of these virulence factors, which may have similar functions in disease establishment [18]. However, *iro*N, *omp*T, *hly*F, *iss*, and *iut*A virulence genes are the most important genes and are reported in more than 70% of APEC isolates [36]. Thus, isolates harboring these virulence genes could develop pathogenic phenotype in chickens.

**Figure-1:**
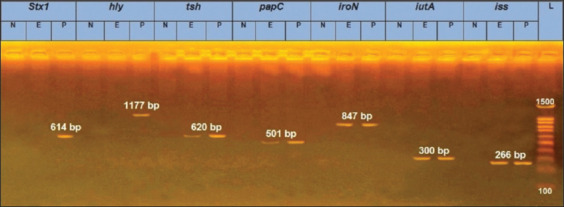
Agarose gel electrophoresis of PCR of seven virulence gene. N=Negative, E=Experiment isolate, P=Positive reference isolate.

In our study, *E. coli* O78 cellulitis-derived isolate from our previous study [10] was screened for seven virulence genes that are commonly associated with pathogenicity. Molecular genotyping revealed that the isolate harbored five virulence genes (*iro*N, *iut*A, *tsh*, *iss*, and *pap*C) but was negative for *stx*1 and *hly* genes (Table-2 and Figure-1). The obtained results indicate that our strain has several of the important VGs that enable its invasion, colonization, evasion from the immune system, and induction of disease. These results were in accordance with Barbieri *et al*. [37], who genotyped 144 cellulitis-derived isolates and recorded that all of them harbored virulence factors related to adhesion, iron acquisition, and serum resistance, which are characteristic of the APEC pathotype. Our selection of these genes as virulence determinants was based on the specific roles they play in the pathogenic process. For instance, the salmochelin siderophore receptor (*iro*N) and ferric aerobactin receptor (*iut*A) are essential for the iron acquisition mechanisms of bacteria, including the production of siderophores that act as ion chelants in the host [38]. It is known that the concentration of free iron in the physiological liquids of animals is low and insufficient for bacterial growth, and pathogenic and invasive bacteria develop high-affinity iron acquisition systems to compete with host siderophores. Of those mechanisms, transferrin ensures bacterial growth in low iron environments. Several studies have confirmed that most APEC strains possess and express the aerobactin iron acquisition system, while non-pathogenic strains produce aerobactin far less frequently [20]. Moreover, the aerobactin system plays a role in the generation and persistence of lesions in APEC-infected chickens [21].

**Table-2:** Gene associated with virulence of the *Escherichia coli* O78 from broiler chicken.

Detected gene	Gene description	Result
*Tsh pap*	Temperature-sensitive hemagglutinin gene.	+
	Pyelonephritis-associated pilli	+
*iron*	Salmochelin siderophore receptor.	+
*iut*A	Ferric aerobactin receptor, iron transporter.	+
*Iss*	Increased serum survival	+
*Hly*	Putative avian hemolysin.	−
*stx*1	Shiga toxin	−

*tsh* is a virulence gene coding for the temperature-sensitive hemagglutinin protein, which is an autotransporter protein possessing the dual functions of adhesive and proteolytic activities. This protein remains in the outer membrane and supports the non-fimbrial adhesion process during the early stages of infection [22]. Furthermore, pyelonephritis-associated pili (*pap*C), a fimbrial adhesion gene, is one of the most important genes contributing to *E. coli* adherence to the host epithelial cells [14,23,28]. The increased serum survival (*iss* gene) of the episome was found to be related to protectins/serum resistance genes, which are responsible for increasing the ability of bacteria to survive in the host serum [18,24].

The previous studies supported our results and, taken together, it can be concluded that the APEC strain is genetically estimated by the presence of at least three to five virulence genes, which enable them to survive an extraintestinal life [15-17]. Moreover, detection of the *papC, iss*, and *tsh* genes is considered to be an important virulence determinant and is detected only in highly pathogenic APEC strains [18,39-43].

The successfully amplified *iro*N, *iss*, and *iut*A genes for *E. coli* isolate were sequenced and submitted to the National Center for Biotechnology Information GenBank, and the accession numbers obtained for *iro*N, *iss*, and *iut*A are MN626681, MN626682, and MN626683, respectively.

**Figure-2:**
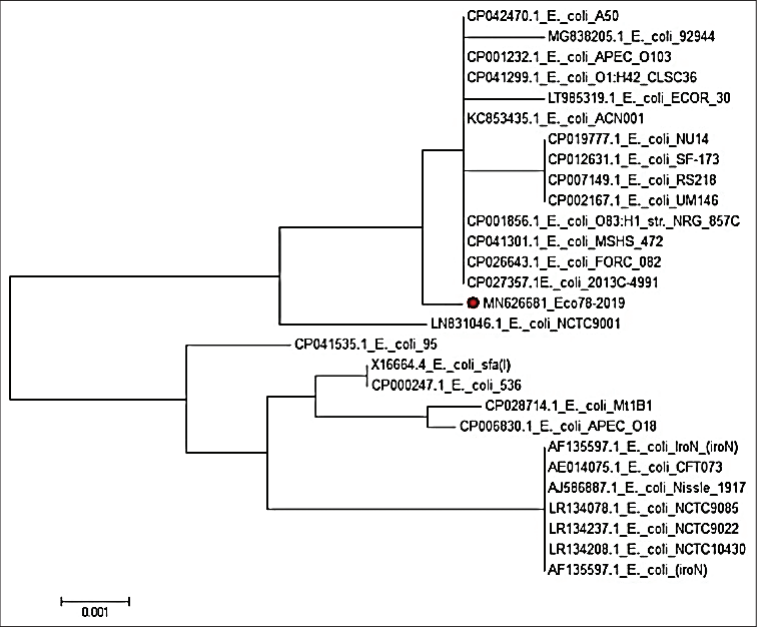
Phylogenetic tree based on the nucleotide sequence of *iro*N gen. Branched distances correspond to sequence divergence.

**Figure-3:**
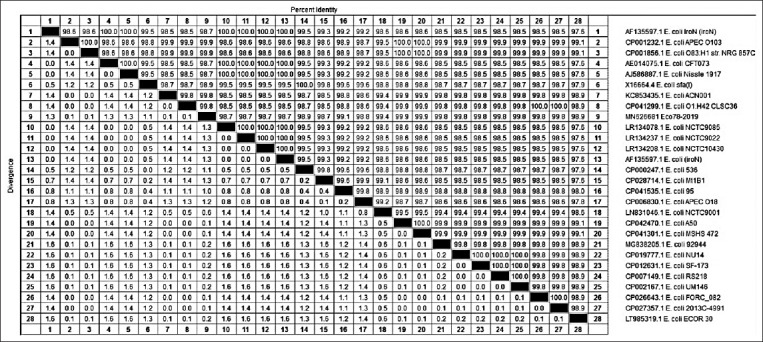
Percentage of nucleotide identities for the *iro*N gen named as MN626681_Eco78- 2019 compared with 27 sequences published in GenBank.

With regard to our phylogenetic analysis of the obtained gene sequences, for the *iro*N gene, our isolate Eco78-2019 showed the highest similarity (99.9%) with isolates *E. coli* APEC 0103, *E. coli* O83:H1 NRG str.857C, *E. coli* A 50, and *E. coli* MSHS 472. In contrast, the lowest similarity (98.7%) with our isolate Eco78-2019 was detected for isolates *E. coli* *Iro*N (*iro*N), *E. coli* CFT073, *E. coli* Nissle 1917, *E. coli* NCTC 9085, *E. coli* NCTC 10430, *E. coli* NCTC 9022, *E. coli* (*iro*N), and *E. coli* Mt 1B1 (Figures-2 and 3).

**Figure-4:**
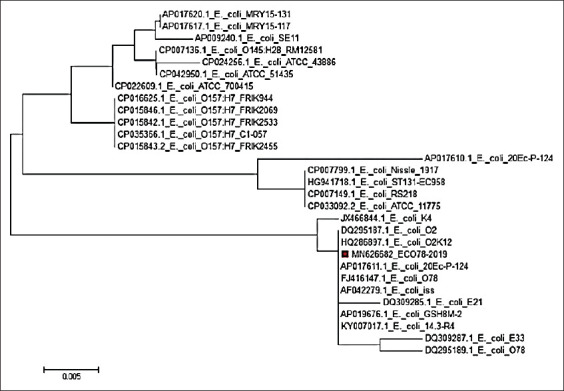
Phylogenetic tree based on the nucleotide sequence of *iss* gene. Branched distances correspond to sequence divergence.

**Figure-5:**
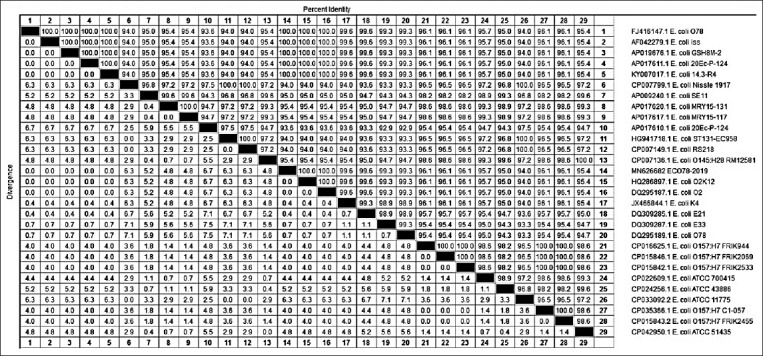
Percentage of nucleotide identities for the *iss* gene named as MN626682_Eco78- 2019 compared with 27 sequences published in GenBank.

For the *iss* gene, our isolate Eco78-2019 showed complete similarity with *E. coli* O78, *E. coli* *iss*, *E. coli* GSH8M-2, *E. coli* 20 Ec-p-124, *E. coli* 14.3-R4, *E. coli* O2k12, and *E. coli* O2. The lowest similarity (93.6%) with our isolate Eco78-2019 was detected for *E. coli* 20 Ec-p-124 (Figures-4 and 5).

**Figure-6:**
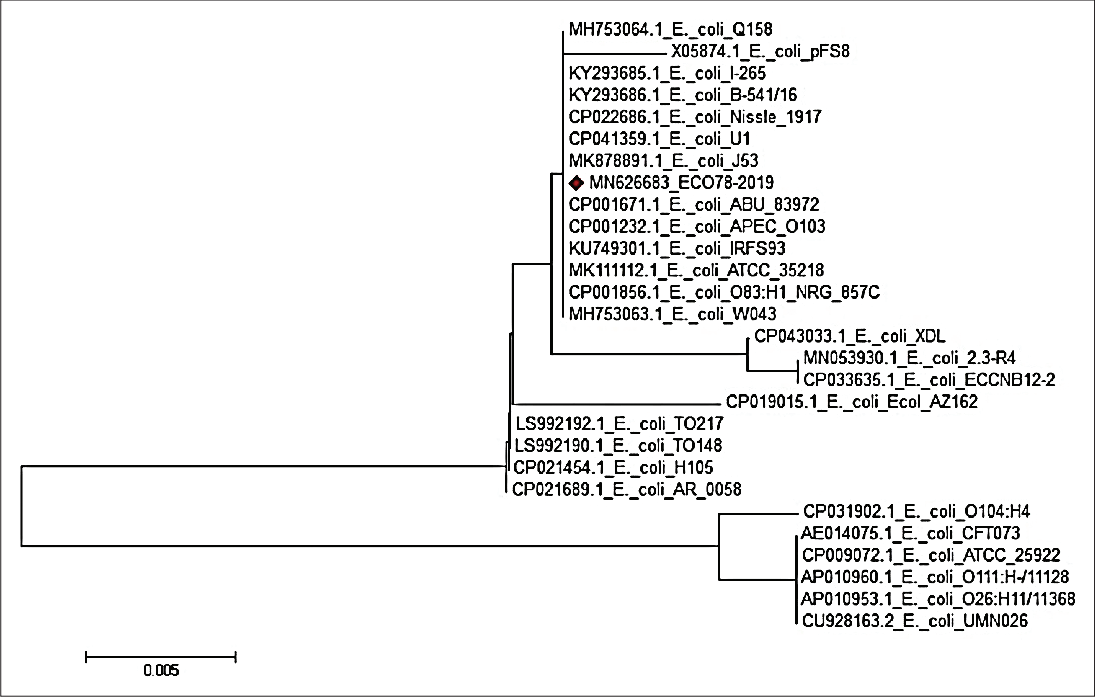
Phylogenetic analysis based on the nucleotide sequence of *iut*A gene. Branched distances correspond to sequence divergence.

**Figure-7:**
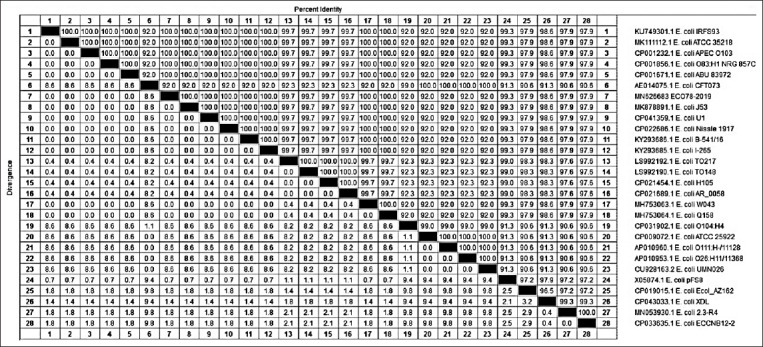
Percentage of nucleotide identities for the *iutA* gene named as MN626683_Eco78- 2019 compared with 27 sequences published in GenBank.

For the *iutA* gene, our isolate showed complete similarity 100% with isolates *E. coli* IRFS93, *E. coli* ATCC 35218, *E. coli* APEC 0103, *E. coli* O83:H1 NRG 857C, *E. coli* ABU 83972, *E. coli* J53, *E. coli* U1, *E. coli* Nissle 1917, *E. coli* B-541/16, *E. coli* I-265, *E. coli* W043, and *E. coli* Q158. The lowest similarity (92%) with our isolate Eco78-2019 was detected for isolates *E. coli* CFT073, *E. coli* O104:H4, *E. coli* ATCC 25922, *E. coli* O111: H-/11128, *E. coli* O26: H11/11368, and *E. coli* UMN026 (Figures-6 and 7).

Analysis of the phylogenetic tree revealed significant similarities between our isolate and the referenced strains. These results are in accordance with Johnson *et al*. [44] and Sedeek *et al*. [45]. These genes are conserved and are not polymorphic; they are rarely all found within an isolate. APEC strains are considered to be heterogeneous bacteria. There is a specific combination of genes for each APEC strain that can cause colibacillosis in poultry [21]. As previous literatures reported, cellulitis might be predisposed by stress conditions, created in and common under field conditions in poultry farms. Therefore, we intended to confirm the pathogenicity of our isolate by estimating its capability to induce cellulitis lesions in chickens raised under minimized stress conditions.

**Figure-8:**
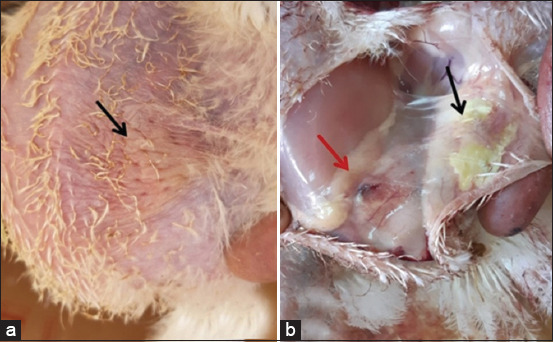
Broiler chicken subcutaneous (SC) infected with *Escherichia coli* on the 3^rd^ DPI. (a) Site of infection showing thick reddish edematous circumscribed lesion (arrow). (b) SC tissue at inoculation site shows edematous thick area with yellowish-white material (black arrow) and the abdominal muscle showing redness and increased vacuolization (red arrow).

In the present study, we observed that chickens inoculated with bacteria showed a low feed intake with ruffled feathers on the 1^st^DPI, followed by red swollen skin and increased skin thickness at the site of injection. On the 3^rd^ DPI, the infected chickens manifested thick red edematous skin with yellowish-white material in the SC tissue at the inoculation site, and the abdominal muscle showed redness and increased vacuolization in 100% of infected birds (Figure-8). Birds with severe lesions were reluctant to move.

**Figure-9:**
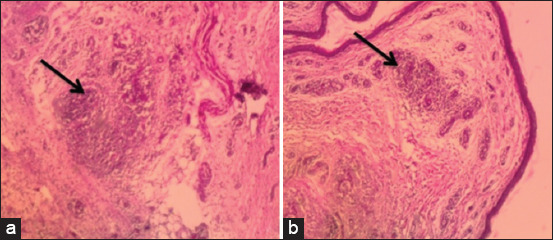
Broiler chicken skin sections on the 3^rd^ DPI with *Escherichia coli*. (a) Subcutis showing caseous inflammation characterized by marked accumulation of heterophils and mononuclear cells (arrow head) in the SC fatty tissue (H and E ×200). (b) Subcutis showing severe infiltration of heterophils and mononuclear cells of the dermis (arrow head) in the SC fatty tissue (H and E ×100).

Histopathological examination of the skin lesions proved to have a moderate-to-severe caseous inflammatory reaction characterized by marked accumulation of heterophils and mononuclear cells in the SC fatty tissue (Figure-9). We recorded four cases of death in the infected group, including two chicks on the 3^rd^ DPI and two chicks on the 5^th^ DPI, with a total of 20% mortalities. *E. coli* was reisolated from the dead and sacrificed birds of the infected group, whereas the first group (the control group) appeared normal and had no obvious clinical abnormalities.

Our clinical study reveals that the postmortem lesions and pathological findings obtained from this experiment confirm the findings of the previous studies. For instance, cellulitis was experimentally induced by the SC inoculation of 25-day-old broiler chickens with a field isolate of *E. coli* serogroup O78, and lesions were detected at 24 h post-infection (PI) in 98% of infected birds with the reisolation of *E. coli* from more than 75% of cellulitis lesions [9]. In another study, cellulitis was induced in 39-day-old broilers in same manner using *E. coli* bacteria. Characteristic cellulitis plaques developed in all infected birds within 18 h PI [6].

The histopathological findings matched the findings of other studies, which reported thickening of the dermis, slight hyperkeratosis, hyperplasia of the epidermis, neovascularization, and infiltration of mononuclear cells and heterophils on microscopic examination of the cellulitis lesions [8]. The previous experimental studies have shown that *E. coli* isolated from a cellulitis origin were more likely to induce cellulitis lesions in experimentally infected birds than were strains of non-cellulitis origin [11]. Furthermore, some cellulitis-derived *E. coli* cause only localized lesions, even when inoculated at high doses [46].

Cellulitis causes great economic losses in the poultry industry, mainly due to an increased condemnation rate and downgrading of affected carcasses [47]. Thus, we targeted our analysis to obtain a rough and brief estimation of the effect of cellulitis on body gain as a proof of economic losses. As a result, the feed intake, average BWG, and FCR were calculated for both the control and infected groups (Table-3). The obtained result revealed that the average feed intake and BWG deceased in infected chickens (5 DPI) (604.35 g and 344.90 g) in comparison with those of the non-infected control chickens (740.60 and 451.80), respectively. Furthermore, the calculated FCR was lower (1.75) for the infected group compared to that of the non-infected group (1.64).

**Table-3:** Average body weight gain, feed intake, and feed conversion rate of control and infected group.

Group	Infection	Age/days	Av.FI/g	ABWG/gm	FCR
1	Control	7	145.69	139.42	1.04
		14	386.75	283.20	1.37
		19	740.60	451.80	1.64
2	*Escherichia coli*	19	604.35	344.90	1.75

Our results confirm the previous studies that demonstrated the economic significance of cellulitis in broiler chickens through its link to increased condemnation rate, downgrading at processing, and costs associated with reprocessing of affected carcasses [5].

## Conclusion

This study proves that molecular techniques can provide an accurate determination of the pathogenicity of virulent bacteria with the advantages of being rapid results, time-saving, and economical. Cellulitis is associated with a lower BWG and decreased FCR, which contributes to economic losses.

## Authors’ Contributions

MMA designedand planned the study, drafted and revised the manuscript. HMM, HSF, AE, MAB, and KME shared in performing the tests, manuscript writing, and data analysis. All authors read and approved the final manuscript.
